# Dynamic Metabolomics and Transcriptomics Analyses for Characterization of Phenolic Compounds and Their Biosynthetic Characteristics in Wheat Grain

**DOI:** 10.3389/fnut.2022.844337

**Published:** 2022-02-16

**Authors:** Dongyun Ma, Beiming Xu, Jianchao Feng, Haizhou Hu, Jianwei Tang, Guihong Yin, Yingxin Xie, Chenyang Wang

**Affiliations:** ^1^College of Agronomy/National Engineering Research Center for Wheat, Henan Agricultural University, Zhengzhou, China; ^2^The National Key Laboratory of Wheat and Maize Crop Science, Henan Agricultural University, Zhengzhou, China; ^3^Henan Technology Innovation Center of Wheat, Henan Agricultural University, Zhengzhou, China

**Keywords:** wheat, phenolics, grain development, metabolomics analysis, transcriptomics analysis

## Abstract

Phenolic compounds are important bioactive phytochemicals with potential health benefits. In this study, integrated metabolomics and transcriptomics analysis was used to analyze the metabolites and differentially expressed genes in grains of two wheat cultivars (HPm512 with high antioxidant activity, and ZM22 with low antioxidant activity) during grain development. A total of 188 differentially expressed phenolic components, including 82 phenolic acids, 81 flavonoids, 10 lignans, and 15 other phenolics, were identified in the developing wheat grains, of which apigenin glycosides were identified as the primary flavonoid component. The relative abundance of identified phenolics showed a decreasing trend with grain development. Additionally, 51 differentially expressed phenolic components were identified between HPm512 and ZM22, of which 41 components, including 23 flavonoids, were up-regulated in HPm512. In developing grain, most of the identified differentially expressed genes involved in phenolic accumulation followed a similar trend. Integrated metabolomics and transcriptomics analysis revealed that certain genes encoding structural proteins, glycosyltransferase, and transcription factors were closely related to metabolite accumulation. The relatively higher accumulation of phenolics in HPm512 could be due to up-regulated structural and regulatory genes. A sketch map was drawn to depict the synthetic pathway of identified phenolics and their corresponding genes. This study enhanced the current understanding of the accumulation of phenolics in wheat grains. Besides, active components and their related genes were also identified, providing crucial information for the improvement of wheat's nutritional quality.

## Introduction

Phenolic compounds are bioactive phytochemicals with one or more aromatic rings and one or more hydroxyl groups, in addition to other substituents (1). These biologically active substances are secondary metabolites in plants and occur in the form of phenolic acids, flavonoids, polyphenolic amides, and other polyphenols derived from the phenylpropanoid (2) or tyrosine pathways (3). Phenolic compounds possess a potent antioxidant nature due to their ability to donate electrons and transfer hydrogen atoms to quench free radicals (4). Flavonoids have higher antioxidant activity than carotenoids or vitamin E (5, 6). Increased consumption of these phytochemicals has been associated with reduced risk of certain chronic diseases, such as obesity, cardiovascular disease, cancer tumor formation, and type-2 diabetes (7). Bioactive phytochemicals have gained a lot of attention due to the increasing demand for natural antioxidant foods.

Wheat (*Triticum aestivum* L.) is cultivated widely worldwide. Apart from rich in carbohydrates and proteins, wheat grains provide significant levels of biologically active substances. According to previous studies, daily consumption of whole wheat grains can provide health benefits and reduce the risk of certain diseases (8, 9). Phenolic acids and flavonoids are the major phenolic compounds in wheat grains (10). The phenolic compounds identified in wheat grains include hydroxybenzoic acids (gallic, protocatechuic, and syringic acids) and hydroxycinnamic acids (ferulic, caffeic, chlorogenic, sinapic, and *p*-coumaric acids) (10). Generally, ferulic acid is the most abundant bound phenolic acid compound accounting for 65.0% of total phenolic acid content (11–13). Flavonoids are also present in wheat grains, and most of them are flavones containing aglycone groups of apigenin, luteolin, or chrysoeriol, and a few of them are flavonols with kaempferol or quercetin as the skeleton structure (14). Phenolic content varies among different wheat cultivars or accessions. A previous study reported that that the inter-genotypic variability of total phenolic content is 18.5% in wheat cultivars (15). Evaluation of phenolic acid content amongst 34 wheat accessions revealed that certain cultivars were rich in phenolic acid content (16). Also, certain old wheat cultivars showed a peculiar phenolic composition with unique nutraceutical values (17, 18). Additionally, a few wheat mutant cultivars with higher flavonoids or cell-bound phenolics were isolated from a wheat ethyl methanesulfonate mutant library (19). Apart from genotypes, environmental factors, such as soil fertilization, rainfall, and temperature, significantly affect the phenolic content of wheat grains (10). The phenolic content and components in wheat grain are different during the grain filling stage. Ultra-performance liquid chromatography-mass spectrometry (UPLC-MS)-based identification of the number of phenolic compounds showed a progressive decline along with the grain development stage (20). A previous study reported that ferulic acid and syringic acid accumulation levels peaked at 14 days after anthesis (DAA) (13).

Phenolic compounds are primarily derived from the phenylpropanoid biosynthetic pathway. In this pathway, the conversion of phenylalanine to cinnamic acid by phenylalanine ammonia-lyase is the first step. Besides, the tyrosine-derived pathway is suggested as another route for the biosynthesis of coumaric acid (2, 3). The enzymes and their encoding genes involved in phenolic biosynthesis have been widely studied, specifically in *Arabidopsis thaliana* and crops, such as maize and rice (21). In a previous expression profiling study, structural genes involved in phenolic biosynthesis and their regulatory genes were associated with the accumulation of phenolic compounds in wheat (13, 22–24). Additionally, several candidate genes involved in the biosynthesis of phenolic components were also identified, and their position on the chromosome was located (19, 25, 26). However, the phenolic components in wheat grains are complex, and the key pathways and regulatory genes for the synthesis of a multitude of phenolic substances remain largely unknown. Many new phenolic components and genes related to phenolic biosynthesis in wheat are yet to be identified. Mutants are crucial for research studies on functional genomics. In this study, the grain during the filling stage from one mutant wheat line with high phenolic content and its wild type control was analyzed to characterize the phenolic components and the corresponding transcription profiling through integrated metabolomics and transcriptomics analysis. The findings in this study will provide new insight into the accumulation of phenolics in developing wheat grains, and the identification of closely related genes regulating key phenolic components biosynthesis.

## Materials and Methods

### Plant Samples

Two winter wheat (*T. aestivum* L.) cultivars (lines), “HPm512” with high antioxidant activity, which was screened from wheat EMS mutant library, and its corresponding wild type cultivar “ZM22,” were analyzed in this study. Wheat seeds were sown during the 2019–2020 growing season at Henan Agricultural University Experimental Station, Zhengzhou, Henan Province, China (34°44′ N, 113°42′ E). The soil characteristics were determined. At 0–20 cm soil depth, the soil organic matter content was 17.47 mg/kg, available phosphorus was 18.07 mg/kg, available potassium was 213.71 mg/kg, total nitrogen was 0.84 g/kg, and pH was 7.91. The wheat seeds were sown on 17 October 2019 with 225 seeds/m^2^. Field trials were managed based on local agronomic practices.

At the flowering stage, wheat spikes of the same size undergoing anthesis on the same day were tagged. The tagged wheat spikes were harvested at 20 DAA, 30 DAA, and maturity. Three biological replicates were sampled for each stage for the two wheat lines. The harvest spikes were immediately frozen in liquid nitrogen and later stored in a −80°C refrigerator.

### Grain Metabolite Extraction and Analysis

The freeze-dried wheat grain samples were crushed using a mixer mill (MM 400, Retsch) with a zirconia bead for 1.5 min at 30 Hz. Approximately 100 mg of powdered wheat grain samples were extracted overnight at 4°C using 1.2 mL of 70% aqueous methanol. The solution was centrifuged for 10 min at 12,000 rpm; the extracts were filtrated (SCAA-104, 0.22 μm pore size; ANPEL, Shanghai, China) before UPLC-MS/MS analysis.

The resulting samples were analyzed using a UPLC-ESI-MS/MS system (UPLC, SHIMADZU Nexera X2; MS, Applied Biosystems 4,500 Q TRAP). The mobile phase consisted of solvent A (pure water with 0.1% formic acid) and solvent B (acetonitrile with 0.1% formic acid). Sample measurements were performed with a gradient program that employed the starting conditions of 95% solvent A and 5% solvent B. Within 9 min, a linear gradient to 5% solvent A and 95% solvent B was programmed, and a composition of 5% solvent A and 95% solvent B was maintained for 1 min. Subsequently, a composition of 95% solvent A and 5% solvent B was adjusted within 1.1 min and maintained for 2.9 min. The column oven was set to 40°C. The injection volume was 4 μL. The effluent was alternatively connected to an ESI-triple quadrupole-linear ion trap (QTRAP)-MS.

Linear ion trap (LIT) and triple quadrupole (QQQ) scans were acquired on a triple quadrupole-linear ion trap mass spectrometer, AB 4,500 Q TRAP UPLC/MS/MS System, equipped with an ESI Turbo Ion-Spray interface, operating in positive and negative ion mode and controlled by Analyst 1.6.3 software (AB Sciex). The electrospray ionization source conditions were: ion source, turbo spray; source temperature, 550°C; ion spray voltage 5,500 V (positive ion mode)/−4,500 V (negative ion mode); ion source gas I, gas II, and curtain gas were set at 50, 60, and 25.0 psi, respectively; the collision gas was high. Instrument tuning and mass calibration were performed with 10 and 100 μmol/L polypropylene glycol solutions in QQQ and LIT modes, respectively. QQQ scans were acquired as Multiple reaction monitoring (MRM) experiments with collision gas (nitrogen) set to medium. Declustering potential (DP) and collision energy (CE) for individual MRM transitions were done with further DP and CE optimization. A specific set of MRM transitions were monitored for each period according to the metabolites eluted within this period.

### Differential Metabolites Selected and KEGG Annotation

Significantly regulated metabolites between different treatments were determined through variable importance for the projection (VIP) ≥1 and absolute log 2FC (fold change) ≥1. VIP values were extracted from the Orthogonal Partial Least Squares-Discriminant Analysis (OPLS-DA) result, which also contains score and permutation plots. These plots were constructed using the R package MetaboAnalystR. Identified metabolites were annotated using the KEGG compound database (http://www.kegg.jp/kegg/compound/).

### RNA-Seq Analysis

RNA from wheat grains sampled at 20 DAA and 30 DAA were extracted using a TRIzol reagent (Invitrogen, Carlsbad, CA, USA) and subjected to RNA-Seq analysis. RNA-Seq analysis was performed by the LC Bio (Zhejiang, China). Each RNA sample had three biological replicates. Raw sequence reads were aligned against the wheat plant genome (ftp://ftp.ensemblgenomes.org/pub/release-5/plants/fasta/triticum_aestivum/dna) using HISAT. GO and KEGG enrichment analyses were performed as described by LC Bio (https://www.lc-bio.cn/). Gene expression levels were identified and normalized using fragments per kilobase of transcript sequence per millions (FPKM) of base pairs sequenced. *P*-value ≤ 0.05 and |log_2_FC|≥1 were defined as thresholds to identify the differentially expressed genes between different test groups.

### Gene Expression Quantification Using Quantitative Real-Time Polymerase Chain Reaction

Total RNA extraction was the same as mentioned above. Reverse transcription was carried out using the RNA First-strand cDNA Synthesis SuperMix (TransScript) as per the manufacturer's instructions. SYBR PrimeScript miRNA RT-PCR Kit was used to perform qPCR reactions on CFX96TM Real-Time System (C1000TM Thermal cycler, BIO-RAD, USA). For qPCR, β*-actin* was used as the internal reference gene to normalize the relative expression levels of candidate genes in the test RNA samples. The primer sequences for qPCR are provided in Supplementary Table 1.

### Gain Characteristic Analyses

Grain protein content was determined with a near-infrared transmittance analyzer (Foss-Tecator 1,241, Höganäs, Sweden), and total starch content was evaluated by using AUTOPOL VI Polarimeter (Rudolph, USA). The total phenolic content, total flavonoid content and antioxidant activity were performed according to the method of Ma et al. (13, 15).

## Results

### Grain Characteristics

HPm512 showed a higher value, and the control cultivar ZM22 showed a lower total phenolic content, total flavonoid content and antioxidant activity (TEAC, DPPH) (Table 1). The difference in these parameters between two cultivars was significant (*P* < 0.05). No significant difference was observed in the weight of grains between these two wheat cultivars (lines). Also, the protein content of HPm512 was significantly higher than that of ZM22.

**Table 1:** Grain characteristics of the two wheat cultivars (lines) HPm512 and ZM22.

**Items**	**TPC** **(mg/g)**	**TFC** **(mg/g)**	**DPPH∙** **(%)**	**TEAC** **(μmol/g)**	**Thousand kernel** **weight (g)**	**Protein** ** content (%)**	**Total starch** **content (%)**
HPm512	4.24 ± 0.01^a^	2.21 ± 0.08^a^	50.85 ± 1.33^a^	8.72 ± 0.38^a^	47.33 ± 1.65^a^	21.91 ± 0.40^a^	60.13 ± 0.23^a^
ZM22	4.02 ± 0.04^b^	2.02 ± 0.05^b^	47.74 ± 0.66^b^	7.70 ± 0.07^b^	47.05 ± 0.07^a^	18.81 ± 0.38^b^	62.33 ± 1.52^a^

*Data represent mean ± standard deviation. Within the same column, mean values followed by different lowercase letters indicate significant differences at P < 0.05 (least significant difference). TPC, Total phenolic content; TFC, Total flavonoid content; TEAC, Trolox equivalent antioxidant capacity; DPPH∙, the 2, 2-diphenyl-1-picrylhydrazyl scavenging capability*.

### Metabolic Profiling of Wheat Grains During the Grain Filling Stage

**Figure 1:**
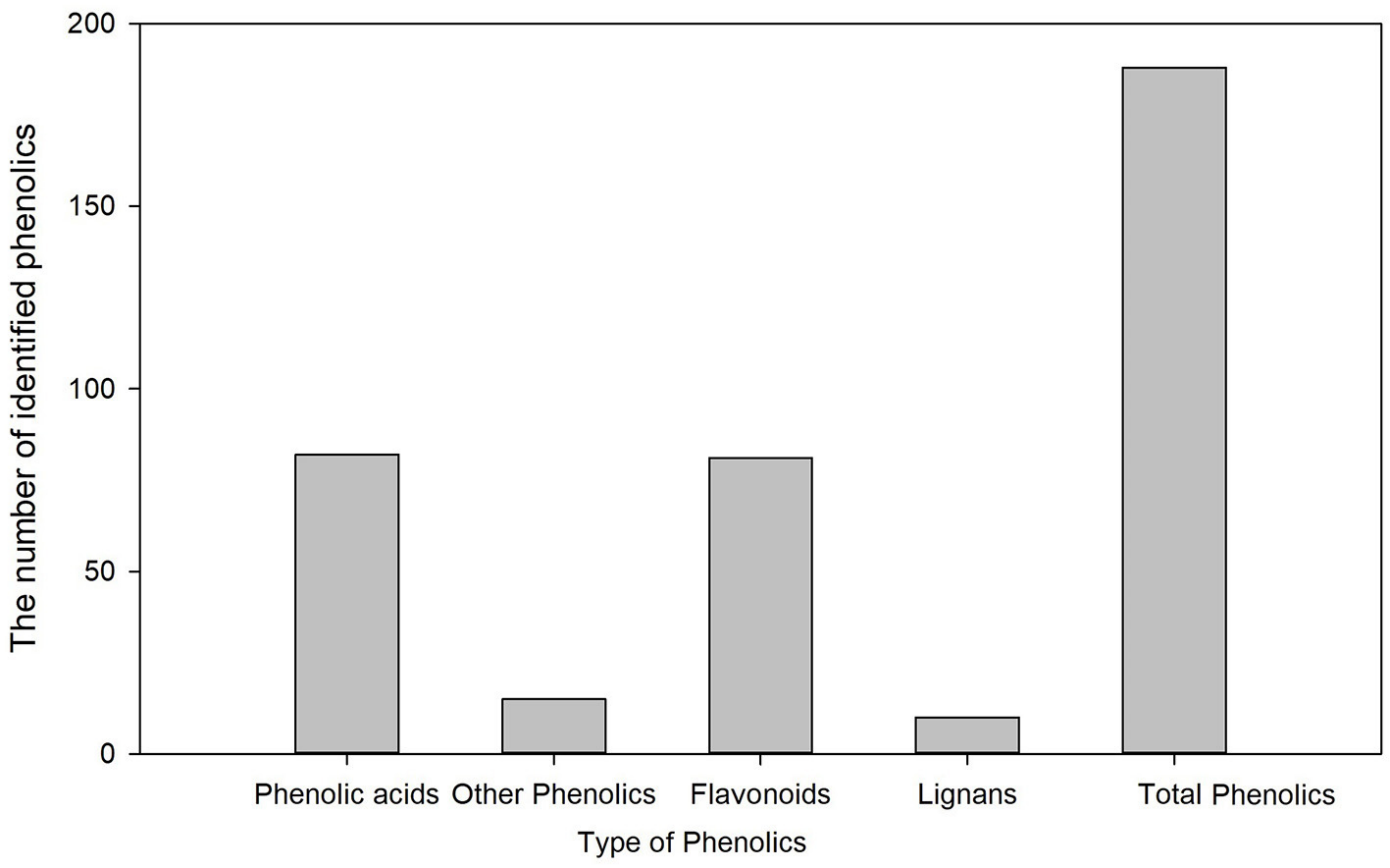
The number of identified phenolics in wheat grains during grain development.

**Figure 2:**
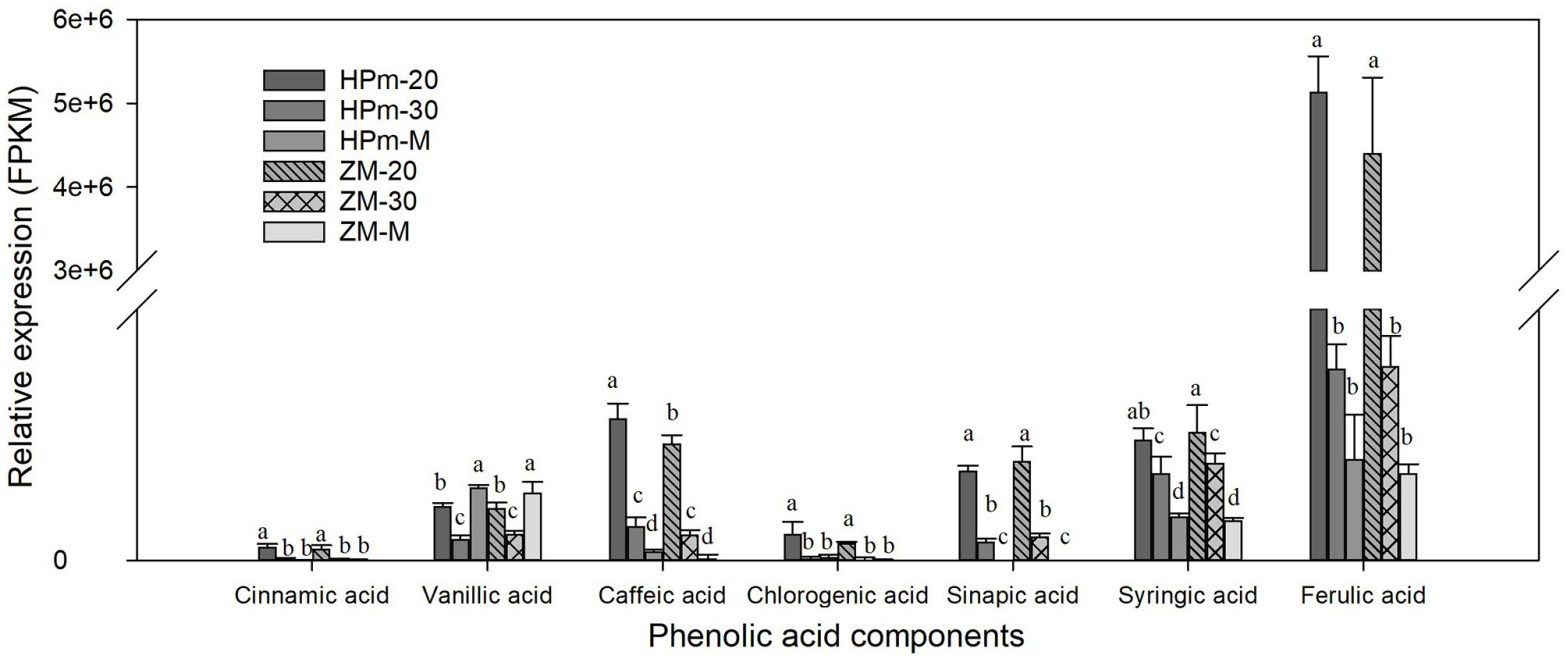
Relative expression levels of identified phenolic acids in wheat grains during grain filling. HPm-20, HPm-30 and HPm-M are samples from wheat cultivar HPm512 at 20 days after anthesis (DAA), 30 DAA, and maturity, respectively. ZM-20, ZM-30, and ZM-M are samples from wheat cultivar ZM22 at 20 DAA, 30 DAA, and maturity, respectively. The different lowcase letters above the column within the same phenolic acid indicate a significant difference (*P* < 0.05).

Metabolic profiling of wheat grain at 20 DAA, 30 DAA, and maturity revealed significant differences in the abundance of phenolics in developing wheat grains (Supplementary Table 2). A total of 188 phenolic components, including 82 phenolic acids, 81 flavonoids, 10 lignans, and 15 other phenolics, were identified in wheat grains during grain development (Figure 1). The relative abundance of most common phenolic acid components in wheat grains, such as ferulic acid, cinnamic acid, chlorogenic acid, syringic, and sinapic acid, showed a decreasing trend with the grain development stage and their levels further decreased at the grain maturity stage (Figure 2). The abundance of ferulic acid in mature wheat grains was about 0.06- and 0.49-fold lower than that of wheat grains at 20 DAA and 30 DAA, respectively. Additionally, ferulic acid showed the highest abundance among these seven phenolic acid components, with a value of 2.77E + 05–3.22E + 05 in mature grains. Certain phenolic acid derivatives, such as 3-O-feruloylquinic acid and trihydroxy cinnamoyl quinic acid with the highest abundance of 3.40 + E05–1.01 + E06 and 1.86 + E06–2.14 + E06 were also identified in mature grains (Supplementary Table 2). Phenolic acid esters, such as methyl ferulate, methyl caffeate, and chlorogenic acid methyl ester, were also identified with a relatively higher abundance in immature grains (Supplementary Table 2). Besides, some phenolic acid components showed relatively higher abundance in immature grains and almost undetectable levels in mature grains. For instance, 5-O-caffeoylshikimic acid showed the relative abundance of 1.42 + E04–1.45 + E04 at 30 DAA but only got the relative abundance of 9 of mature grain. The high abundance of phenolic acid components in immature grains suggests that immature grains may be a rich source of antioxidants. Some phenolic acid glycosides, such as salicylic acid-2-O-glucoside and *p*-coumaric acid-4-O-glucoside, were also identified (Supplementary Table 2).

**Figure 3:**
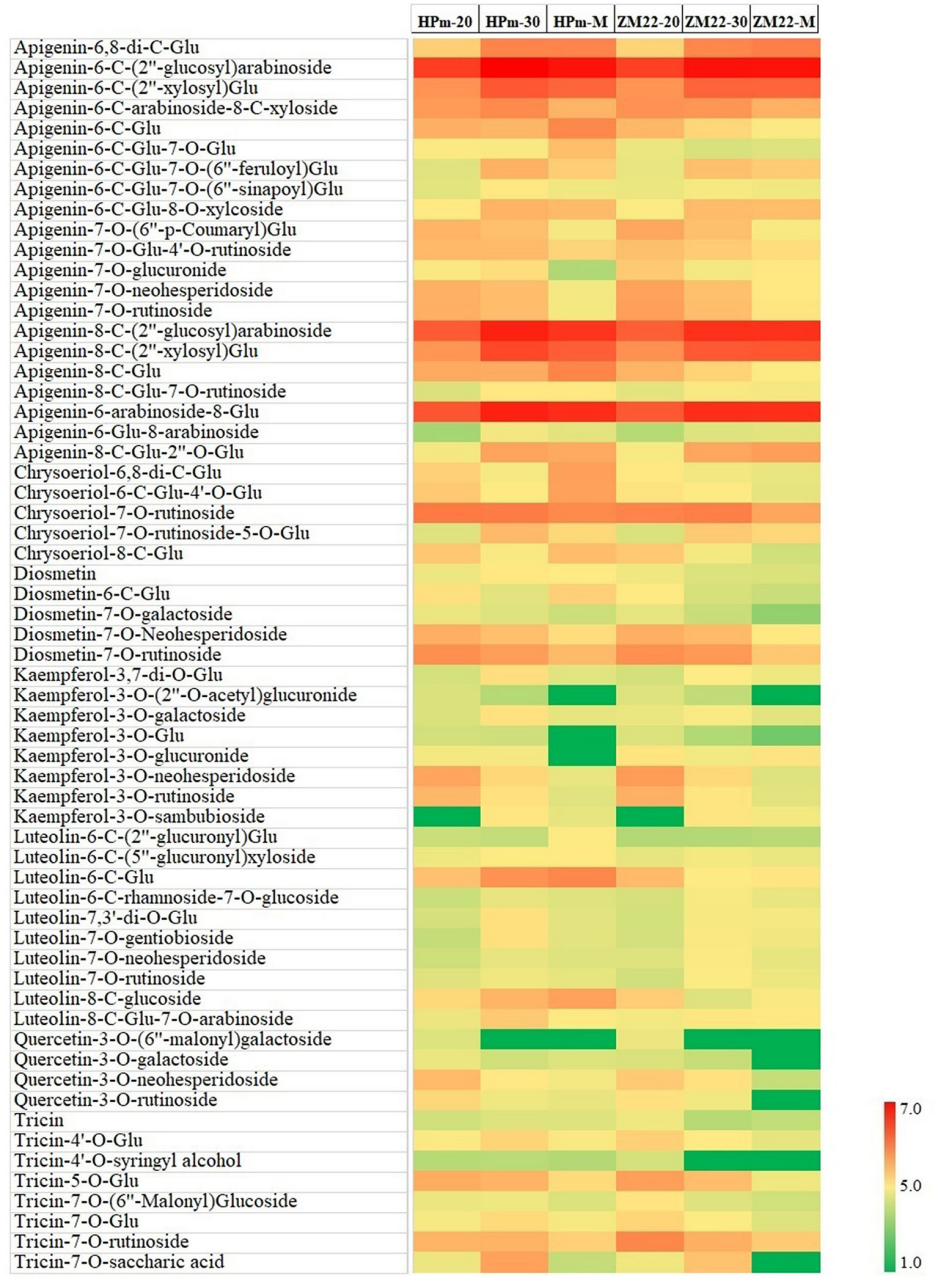
Heat map of differentially expressed flavonoids in wheat grains during grain development. HPm-20, HPm-30, and HPm-M are samples from wheat cultivar HPm512 at 20 days after anthesis (DAA), 30 DAA, and maturity. ZM-20, ZM-30, and ZM-M are samples from wheat cultivar ZM22 at 20 DAA, 30 DAA, and maturity, respectively. Red color represents high relative abundance, and green color represents low relative abundance.

In this study, a total of 81 flavonoid components were identified, mainly in the form of glycosides, including apigenin, luteolin, kaempferol, and quercetin type. A heat map of differentially expressed flavonoids in wheat grains was also constructed (Figure 3). Out of these differentially expressed flavonoids, apigenin glycosides were the most abundant flavonoid components. A total of 21 differentially expressed apigenin glycosides were identified, of which apigenin-6-C-(2″-glucosyl)-arabinoside and apigenin-8-C-(2″-glucosyl)-arabinoside had a high abundance of 3.54 + E06–7.59 + E06, while apigenin-6-C-glucoside-7-O-(6″-sinapoyl)-glucoside and apigenin-7-O-glucuronide showed a low level of abundance of 3.14 + E03–2.63 + E04. In addition, ten luteolin glycosides were also identified, specifically for luteolin-6-C-glucoside, which showed the highest abundance of 4.63E + 05 and 4.86E + 04 at HPm512 and ZM22 mature grains, respectively. Tricin and seven tricin glycosides were found, of which tricin-7-O-rutinoside had the highest abundance of 7.97 + E04–9.18 + E04 in mature grains. Kaempferol and quercetin belong to flavonols, which generally have high antioxidant activity. In this study, eight kaempferol glycosides were identified, of which kaempferol-3-O-neohesperidoside and kaempferol-3-O-sambubioside showed a relatively higher abundance, while kaempferol-3-O-glucoside and kaempferol-3-O-glucuronide showed a lower abundance. Four quercetin glycosides were identified, and their relative abundance in mature grains was found to be low. Also, five diosmetin glycosides and five chrysoeriol glycosides were identified in this study. The abundance of the majority of the identified flavonoid components showed a decreasing trend from 20 DAA to maturity.

### Differentially Expressed Metabolites Between the Two Wheat Lines

**Figure 4:**
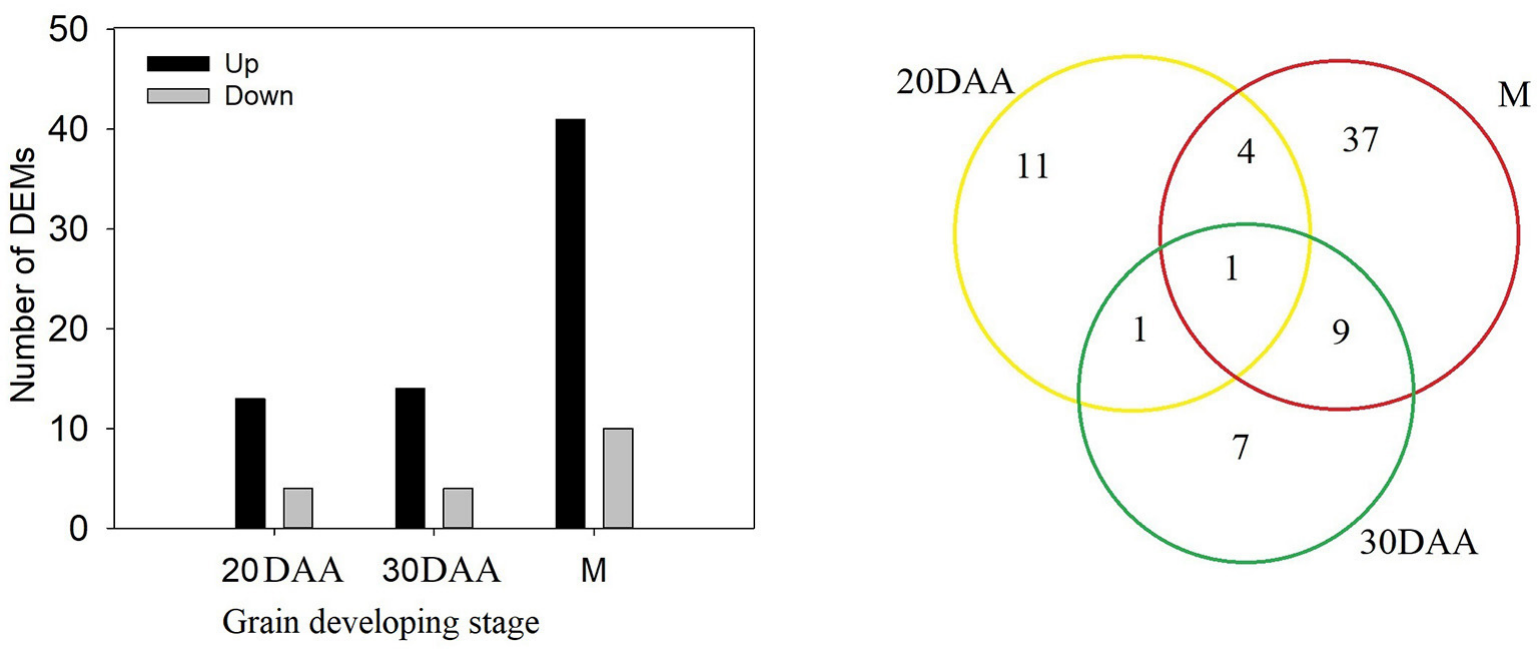
The number of differentially expressed metabolites (DEMs) between HPm512 and ZM22 at 20 days after anthesis (DAA), 30 DAA, and maturity (M). Yellow, green, and red circles indicate 20 DAA, 30 DAA, and maturity.

Comparison of phenolic components in HPm512 and ZM22 revealed that 17 phenolic components, including 7 phenolic acids and 18 phenolic components (including 9 flavonoids), were significantly and differentially expressed at 20 DAA and 30 DAA, respectively (Figure 4). Out of these differentially expressed phenolic components, the number of up-regulated phenolic components in HPm512 was 13 and 14 of 20 DAA and 30 DAA, respectively. In addition, 51 significantly expressed phenolics were detected in mature grains, of which 41 were up-regulated in HPm512, indicating that there were more differentially expressed phenolics in mature grains (Supplementary Table 3). The relative abundance of caffeic acid, 3-O-feruloylquinic acid, and 3,4-dimethoxycinnamic acid in HPm512 was increased by 3.86-, 2.97-, and 276.89-fold, respectively, compared to ZM22. Additionally, the relative abundance of chlorogenic acid methyl eater, coniferin, and *p*-coumaryl alcohol in HPm512 was increased by 400.3-, 3306.2-, and 469.8-fold, respectively, than in ZM22 mature grains. Furthermore, for other common presented phenolic acid components in wheat grains, such as ferulic acid, vanillic acid, syringic acid, and chlorogenic acid, HPm512 got a higher abundance while ZM22 got a lower value. But the difference of the relative abundance between the two wheat cultivars was not significant.

**Figure 5:**
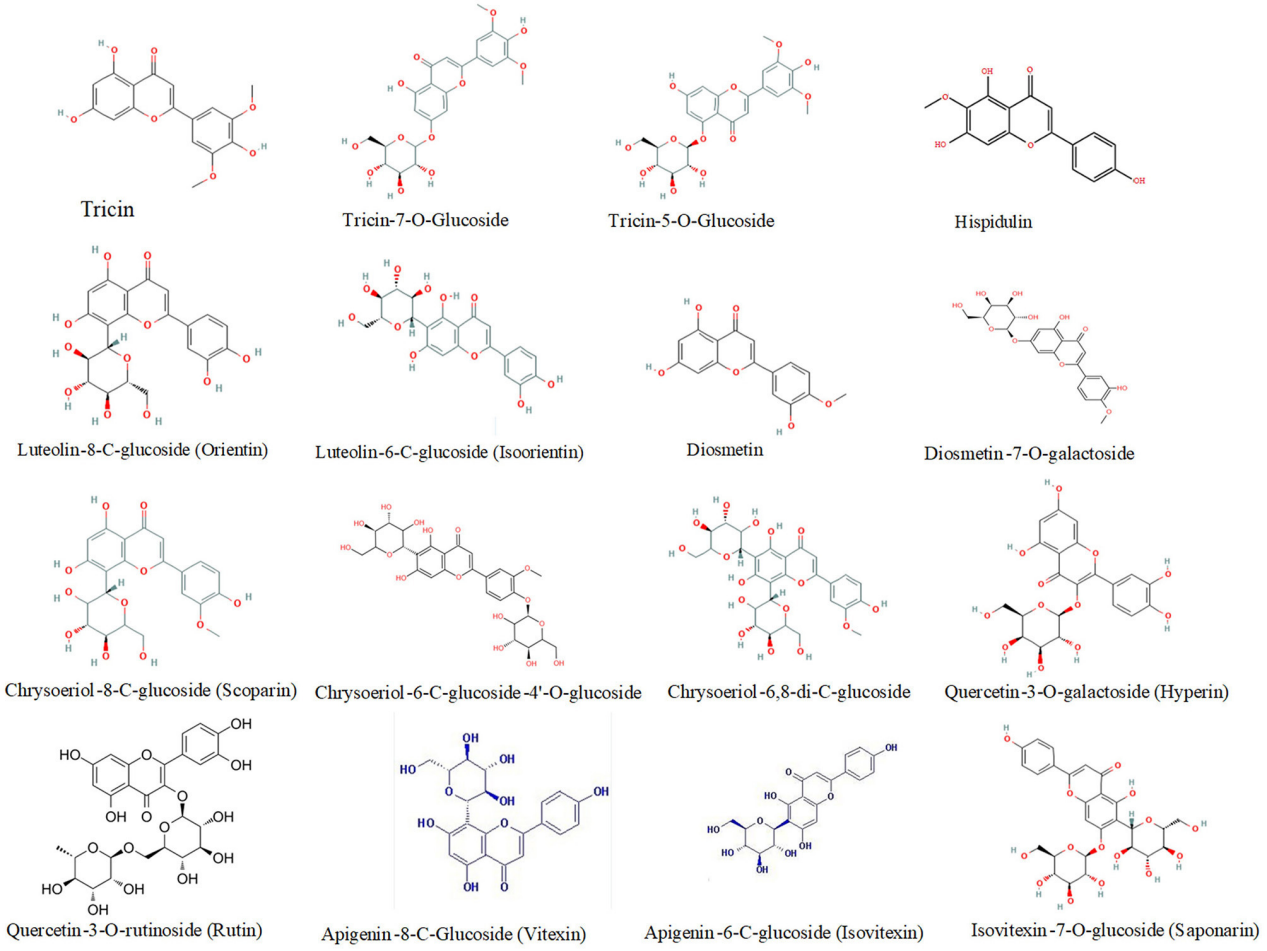
The chemical structure of differentially expressed flavonoid components between HPm512 and ZM22.

Compared to phenolic acid components, a higher number of DEMs belonged to flavonoids, especially at the maturity stage. These flavonoid components were mainly tricin, luteolin, quercetin, hispidulin, diosmetin, chrysoeriol, and their derivatives. For instance, the relative abundance of five tricin-type flavonoid compounds, such as tricin, tricin-4'-O-syringyl alcohol, tricin-7-O-saccharic acid, tricin-7-O-glucoside, and tricin-5-O-glucoside was 2.52, 393.7-, 667-, 2.21-, and 2.28-fold higher, respectively, in HPm512 than in ZM22. The relative abundance of luteolin-8-C-glucoside (orientin) and luteolin-6-C-glucoside (isoorientin) was 3.53E + 04 and 4.86E + 04, in ZM22, and 24.41E + 04 and 46.33E + 04 in HPm512, respectively. Furthermore, the relative abundance of two quercetin glycosides, quercetin-3-O-rutinoside (rutin) and quercetin-3-O-galactoside (hyperin) was found to be 1.94E + 04 and 1.46E + 04, respectively, in HPm512, whereas their abundance in ZM22 was almost undetectable. High antioxidant activity of HPm512 may be attributed to the up-regulation of these phenolic components. The chemical structure of candidate up-regulated flavonoids in HPm512 mature grains is demonstrated in Figure 5.

### Transcriptome Analyses of HPm512 and ZM22 Wheat Grains at the Filling Stage

Wheat grains of HPm512 and ZM22 at 20 DAA and 30 DAA were sampled, and 12 complementary DNA (cDNA) libraries were constructed. After RNA-Seq analysis, the uniquely mapped reads ranged from 30,905,732 to 35,411,432, representing 62.66–70.32% of the total reads (Supplementary Table 4). The number of non-spliced reads ranged from 28,517,743 to 29,409,701, representing 57.96–59.11% of the total reads.

**Figure 6:**
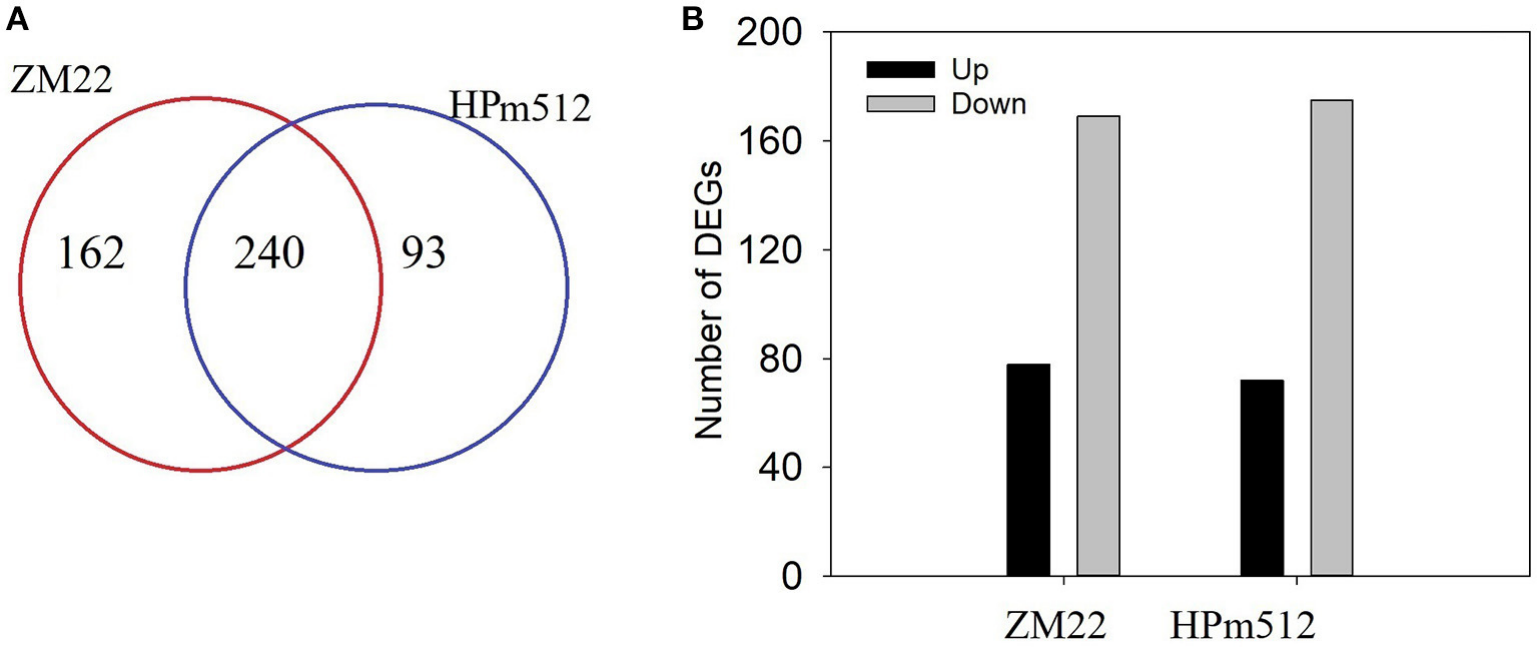
The number of DEGs in wheat grains at 20 DAA and 30 DAA. **(A)** The number of total DEGs involved in phenolic biosynthesis, glycosyltransferase, and transcription factor activity. **(B)** The number of DEGs shared by ZM22 and HPm512 (20 DAA vs. 30 DAA).

In this study, transcripts involved in phenolic accumulation in wheat grains were primarily focused. Thus, the genes encoding the key enzyme involved in the phenolic biosynthesis pathway and transcription factors were primarily targeted. A total of 402 differentially expressed genes (DEGs) involved in phenolic accumulation were detected in ZM22, whereas 333 DEGs were identified in HPm512 during the grain filling stage (Figure 6). Out of these DEGs, 240 were common between HPm512 and ZM22, of which 234 DEGs followed an identical trend from 20 DAA to 30 DAA, and 69 was up-regulated and 165 were down-regulated (Supplementary Table 5). The expression pattern of the majority of down-regulated genes followed a similar trend with decreasing relative abundance of phenolic components during the grain filling stage. Out of these down-regulated structural genes involved in phenolic biosynthesis, 10 genes encode cinnamyl alcohol dehydrogenase, 16 genes encode flavonol synthase, and 13 genes encode cinnamoyl-CoA reductase. In addition, 41 DEGs encoding glycosyltransferase included 27 down-regulated and 13 up-regulated genes from 20 DAA to 30 DAA. Out of the 13 up-regulated genes from 20 DAA to 30 DAA, 8 genes encode flavonol synthase, 6 encode cinnamoyl-CoA reductase, and 7 encode shikimate O-hydroxycinnamoyltransferase. Different expression profiles of homologous genes suggest that they may play a different role in phenolic biosynthesis. The expression levels were remarkably different among these homologous. For instance, out of the genes encoding flavonol synthase, the expression levels of TraesCS4B02G374300 and TraesCS7B02G042500 were higher, while the expression levels of TraesCS7A02G140200 and TraesCS5D02G565100 were lower. These highly expressed homologous genes may play a more important role in phenolics accumulation.

In total, 83 DEGs were identified in HPm512 as compared to ZM22 at 20 DAA. A total of 72 genes were up-regulated in HPm512. Out of these 72 genes, 15 genes encode corresponding structural proteins, 15 encode glycosyltransferase, and 42 encode transcription factors (Supplementary Table 6). Among these structural genes involved in phenolic biosynthesis, 2 genes encoding cinnamoyl-CoA reductase (TraesCS7A02G396100 and TraesCS7B02G298300) were up-regulated in HPm512, of which TraesCS7A02G396100 showed a higher expression level. Two genes encoding flavonol synthase (TraesCS1A02G221200 and TraesCS1A02G320000) also showed a higher expression level in HPm512, of which TraesCS1A02G320000 showed the highest expression level with the FRKM of 15.40. Out of these transcription factors, TraesCS6A02G335400 and TraesCS1D02G230900 showed the highest expression levels with the FRKM of 9.67 and 8.68, respectively, in HPm512 at 20 DAA. Two other transcription factors, TraesCS6A02G229500 and TraesCS4B02G186900, also showed a higher expression level of 4.49- and 4.10-fold, respectively in HPm512 compared to ZM22. There were 14 DEGs involved in ethylene-responsive transcription, of which 13 were up-regulated in HPm512.

At 30 DAA, 249 DEGs were identified in HPm512 compared to ZM22. Out of these 249 DEGs, 40 DEGs encode glycosyltransferase, 85 encode structural proteins, and 124 encode transcription factors (Supplementary Table 6). Unlike the expression pattern at 20 DAA, a higher number of genes were down-regulated in HPm512, which entailed 52 structural proteins, 27 glycosyltransferase, and 89 transcription factor encoding genes. Twelve DEGs encoding flavonol synthase were also identified, of which 5 were up-regulated, and 7 were down-regulated in HPm512. Although the number of down-regulated genes was more than that of up-regulated genes, the up-regulation extent of up-regulated genes was large. For example, the up-regulated genes, TraesCS7B02G041600 and TraesCS7B02G041900, showed remarkable expression levels of 14- and 96-fold increase in HPm512, respectively. TraesCS4B02G081200, a gene encoding flavone synthase, showed a 4.77-fold increase in HPm512 compared to ZM22. Additionally, three genes encoding phenylalanine ammonia-lyase also showed a higher expression level in HPm512. Out of these DEGs, 31 were identified as an ethylene-responsive transcription factor, which included 27 down-regulated genes in HPm512. Fifteen bZIP transcription factors were differentially expressed in HPm512 compared to ZM22.

### qPCR-Based Validations of Identifying Genes

**Figure 7:**
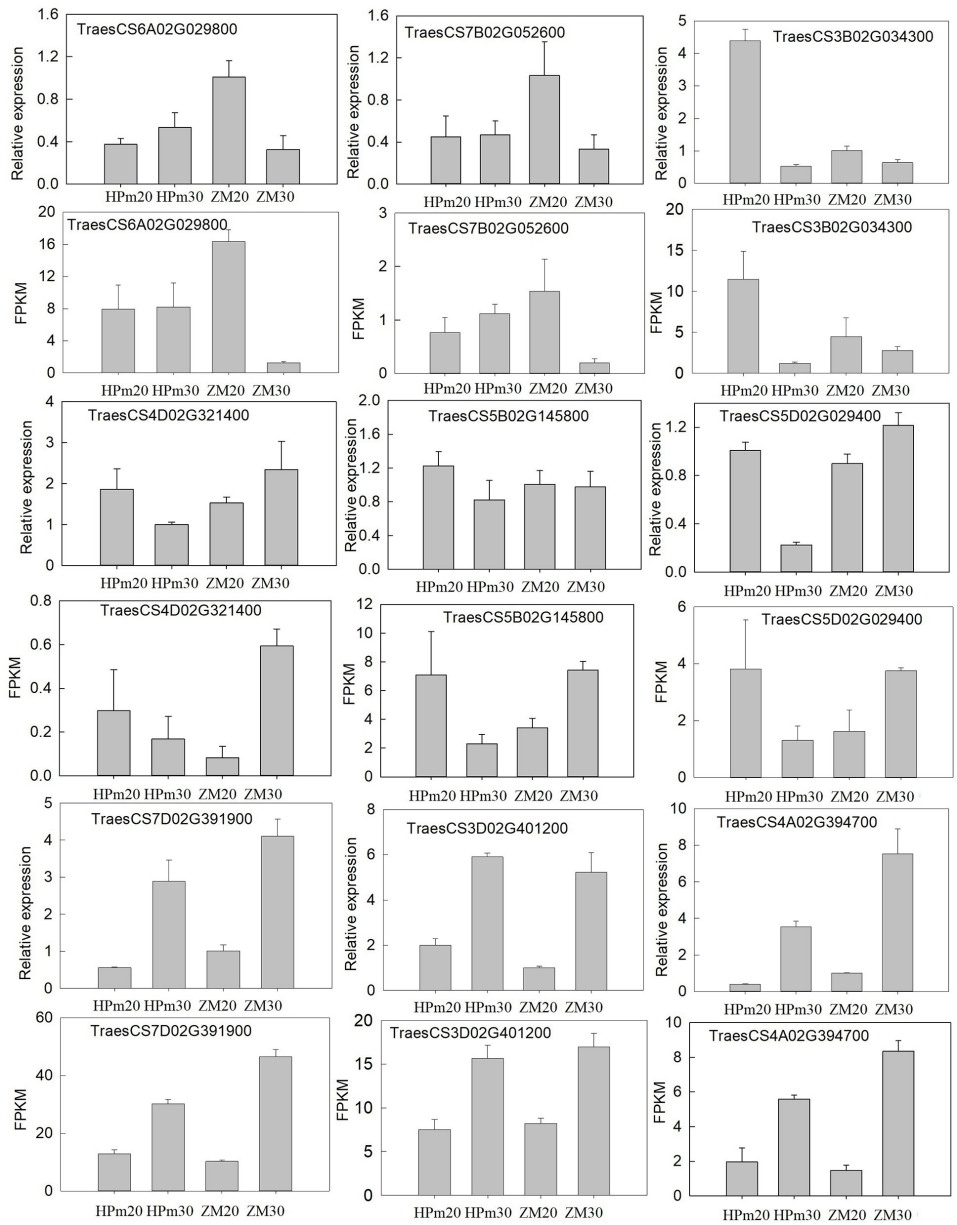
Validation of the expression patterns of nine representative DEGs during the grain filling stage. Relative expression level determined using real-time PCR; FPKM determined using RNA-Seq. HPm20 and HPm30 are samples from HPM512 at 20 and 30 DAA; ZM20 and ZM30 are samples from cultivar ZM22 at 20 and 30 DAA, respectively.

To confirm the expression of genes and validate the deep sequencing results, nine genes were randomly selected for qPCR-based validation. The relative expression levels of these genes were determined using qPCR, and the FPKM tested by deep sequencing were compared to the results of qPCR analysis (Figure 7). The expression patterns of these nine genes in HPm512 and ZM22 at 20 DAA and 30 DAA were similar to those obtained from deep sequencing, suggesting that the RNA-Seq analysis results obtained in this study are reliable.

### Combined Transcriptomics and Metabolomics Analysis

**Figure 8:**
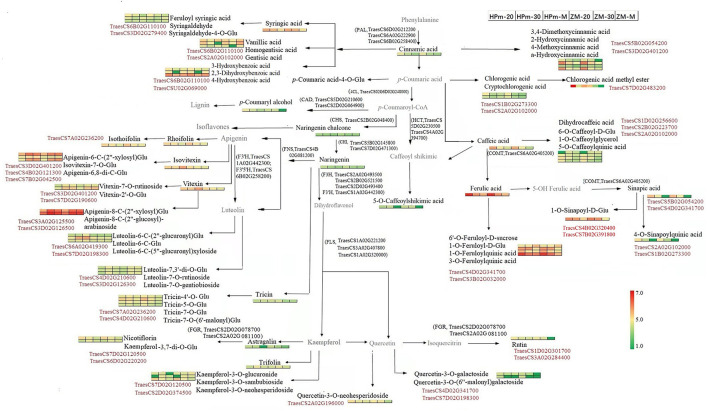
Schematic map of phenolic biosynthesis pathway involved in wheat grains during the grain filling stage. The 1 × 6 heat map represents normalized relative metabolite abundance of grains at a different stage. Red color represents higher abundance, and green color represents lower abundance. HPm-20, HPm-30, and HPm-M are samples from cultivar HP512 at 20 days after anthesis (DAA), 30 DAA, and maturity, respectively. ZM-20, ZM-30, and ZM-M are samples from cultivar ZM22 at 20 DAA, 30 DAA, and maturity, respectively. Metabolites in black color represent significantly expressed metabolites. The gene number in black color represents the biosynthesis process, which is catalyzed by the enzyme encoded by this gene. The gene number in red color represents a significant correlation between this gene and its nearby metabolite. CAD, Cinnamyl alcohol dehydrogenase; CHS, Chalcone synthase; CHI, Chalcone isomerase; 4CL, 4-Coumarate-CoA ligase; FGR, Flavonol−3-O gucoside L-rhamnosyltransferase; FNS, Flavone synthase; COMT, Caffeate-O-methyltransferase; F3H, Flavanone 3-hydroxylase; F3′H, Flavonoid 3-monooxygenase; F3′5′H, Flavonoid 3′,5′-hydroxylase; FLS, Flavonol synthase; PAL, Phenylalanine ammonialyaise.

An integrated metabolomics and transcriptomics data analysis was performed using KEGG (http://www.kegg.jp/kegg/compound/) metabolic pathways. A total of 298 DEGs and 6 DEMs were enriched in the phenylpropanoid biosynthesis pathway, whereas 117 DEGs and 4 DEMs were enriched in flavonoid biosynthesis (Supplementary Table 7). The DEMs enriched in the flavonoid pathway were primarily naringenin and its derivates (naringenin chalcone, naringenin-7-O-glucoside, and naringenin-7-O-neohesperidoside). In addition, 24 DEGs and 5 DEMs, including kaempferol-3-O-galactoside, luteolin-7-O-neohesperidoside, vitexin-2″-O-glucoside, kaempferol-3-O-rutinoside, and quercetin-3-O-rutinoside, were enriched in flavone and flavonol biosynthesis pathways. Enrichment of a higher number of DEGs in these pathways suggests that there may be a multi-gene cooperative model for phenolic biosynthesis. Based on the DEMs and DEGs identified in this study, a sketch map was drawn to illustrate the synthesis pathways of phenolic compounds in the grain (Figure 8). Three genes encoding phenylalanine ammonialyase, which are required for the first step for the phenylpropanoid biosynthesis pathway, were also identified in this study. Several homologous genes encoding flavonol synthases, such as TraesCS1A02G221200 and TraesCS1A02G320000, could be related to the synthesis of flavonol components. In addition to the known structural genes involved in phenolic biosynthesis, genes encoding glycosyltransferase and transcription factors were closely related to the identified metabolites, as shown in Figure 8. For instance, the expression level of the transcription factor TraesCS7D02G120500 was closely related to kaempferol-3-O-neohesperidoside and kaempferol-3-O-rutinoside (nicotiflorin).

In order to explore the key regulatory and structural genes for the high antioxidant activity in HPm512, the correlation between DEMs at maturity and DEGs at 20 DAA and 30 DAA was analyzed using Pearson correlation (Supplementary Tables 8, 9). The correlation network was plotted using Cytoscape v.3.6.0 (www.cytoscape.org/) (Supplementary Figures 1, 2) and correlation coefficient |r|≥0.85 and *P* < 0.05. TraecCS7D02G198300, encoding glycosyltransferase, was highly and positively correlated with hyperin (*r* = 0.99), tricin-7-O-glucoside (*r* = 0.99), diosmetin-6-C-glucoside (*r* = 0.95), chrysoeriol-6-C-glucoside-4'-O-glucoside (*r* = 0.95), vitexin (*r* = 0.89), and isovitexin (*r* = 0.89) (Supplementary Figure 1A). Also, another glycosyltransferase gene (TraesCS1D02G301700) was highly and positively correlated with rutin (*r* = 0.95), diosmetin-7-O-galactoside (*r* = 0.95), and tricin-7-O-saccharic acid (*r* = 0.85). For structural genes involved in the phenolic biosynthesis pathway, two chalcone isomerase genes (TraesCS5B02G145800 and TraecCS02G145400) were positively correlated with tricin-7-O-glucoside, hyperin, luteolin-6-C-(5″-glucuronyl) xyloside, and rutin. In addition, the transcription factor TraesCS3D02G244300 was positively correlated with several flavonoids, such as vitexin (*r* = 0.98), isoorientin (*r* = 0.98), orientin (*r* = 0.97), isovitexin (*r* = 0.99), and scoparin (*r* = 0.97). A positive correlation was observed between rutin and several transcription factors encoding genes, such as TraesCS3A02G284400 (*r* = 0.97) and TraesCS2A02G505600 (*r* = 0.95), which indicate that the biosynthesis of rutin may be related to the coordinated regulation of multiple genes. Similarly, the correlation coefficients between DEMs and DEGs at 30 DAA also revealed that there might be synergistic or antagonistic effects among the DEGs. For example, rutin was positively correlated with TraesCS4A02G107400 and TraesCS3D02G502000, but negatively correlated with TraesCS5B02G436400 and TraesCS6D02G407000. TraesCS5B02G115100, encoding a MADS-box transcription factor, was positively correlated with rutin (*r* = 0.95), luteolin-6-C-(5″-glucuronyl) xyloside (*r* = 0.97), and diosmetin-7-O-galactoside (*r* = 0.95). Also, two bZIP transcription factors encoding genes (TraesCS4B02G320400 and TraesCS7B02G391800) were positively correlated with 1-O-galloyl-D-glucose, 1-O-sinapoyl-D-glucose, and protocatechuic acid-4-O-glucoside.

## Discussion

The grain filling stage is important for grain development and the accumulation of various nutritional components. Kernel morphology, dry matter content, and antioxidant content vary with the grain development stage. Phenolic concentration, determined using the dry weight of grain, decreases as the grain matures (27). The levels of most of the tested phenolic acids, including caffeic acid and ferulic acid, declined with grain development, and their peak values were observed at 7 DAA or 14 DAA (13). In this study, the most common phenolic acid components in wheat grains, such as ferulic acid, cinnamic acid, and chlorogenic acid, showed a decreasing abundance with grain development. Their lowest abundance values were observed at the maturity stage of the grain. In addition, the relative levels of most of the identified flavonoid compounds declined during grain development. This finding is in line with the study by Santos et al. (20). The reduced level of the phenolic antioxidants could be attributed to the dilution of the dry matter (28, 29) and the decrease of assimilate availability for phenolic biosynthesis in the middle and later grain filling stages (30). It is noteworthy that specific phenolic components, such as luteolin-8-C-glucoside (orientin) and luteolin-6-C-glucoside (isoorientin), showed a higher expression level in mature grains compared to immature grains. The increase in the level of these flavonoid components may result from their higher rate of synthesis in the later grain filling stage, for polyphenol compound is regulated by the balance of its biosynthesis and degradation (31). The high phenolic content in immature grains suggests that immature grains, such as grains from the late milk stage and dough stage, can be used as raw materials for nutritious food.

More than 500 phenolic components have been reported in foodstuffs (32). A total of 237 phenolic components, including 85 flavonoids, 77 phenolic acids, 51 other polyphenols, 16 lignans, and 8 stilbenes, were identified in different developmental stages of wheat grain (20). In this study, a total of 188 phenolic components, including 82 phenolic acids and 81 flavonoids, were identified, and their relative abundance changed significantly with grain development. Generally, ferulic acid, cinnamic acid, syringic acid, and caffeic acid are the most abundant phenolic acids in wheat grains (33, 34), of which ferulic acid is the most abundant (35). Here, among the identified phenolic acids, ferulic acids showed the highest abundance. Several phenolic acid esters, including chlorogenic acid methyl ester, have also been identified with a relatively higher abundance in mature grains. Accumulation of these phenolic esters might improve wheat grain's anti-inflammatory properties, as it was reported that these phenolic esters possess strong anti-inflammatory activity by inhibiting the pro-inflammatory cytokines (36, 37).

The majority of identified flavonoids by Liu et al. (14) were classified as flavones with aglycone groups of luteolin, apigenin, or chrysoeriol, and a few were identified as flavonols with kaempferol or quercetin as the backbone. In this study, most of the identified flavonoids were classified as flavones (21 with aglycone groups of apigenin, 10 of luteolin, and 4 of chrysoeriol). These results indicate that most of the flavonoid components in common wheat grains are flavones. Furthermore, out of the DEMs identified during the grain filling stage, 27 showed C-glycosidic forms, and 46 showed O-glycosidic forms, which are in line with the previous study stating that most of the natural flavonoids are O-glycosides with sugar bonds (38).

Phenolic components and their content vary between different wheat lines (33, 39), and screening phenolic-rich wheat lines would enhance wheat grain antioxidant potential (19). In this study, 15 phenolic acids and 23 flavonoid components were significantly up-regulated in HPm512 compared to the control cultivar ZM22. For some flavonoid components, such as rutin and hyperin, the relative expression level was almost undetectable in ZM22, whereas it was relatively high in HPm512. Many flavonoid components, such as isoorientin and hyperin, have the potential to ameliorate diverse metabolic complications (40, 41). In this study, the relative expression level of orientin and vitexin in HPm512 was 6.83- and 12.74-fold higher than that in ZM22. These highly expressed bioactive substances suggest that HPm512 may have significant antibacterial or anti-inflammatory activity. Thus, it should be further validated in animal models. Furthermore, for most common phenolic acids presented in wheat gains, such as ferulic acid and chlorogenic acid, the relative abundance of these phenolic acids in HPm512 was higher than that in the control, but the difference was not significant. One explanation of this was the accumulation of phenolic acid derivatives. For example, the relative abundance of chlorogenic acid methyl ester and 3,4-Dimethoxycinnamic acid in HPm512 was 440.27- and 276.98-fold higher than that in ZM22. These highly expressed phenolic acid derivatives are bioactive substances with potential health benefits (37, 42), which may enhance the nutritive value of HPm512.

Also, it may be caused by differences in test methods. Widely targeted metabolome enables the identification of more phenolic compounds, but the detected metabolites are relatively quantitative. It is widely accepted that the antioxidant activities of phenolic compounds were significantly different (10, 43). Exploring the substances with high bioactive components in HP512 and verifying their activity *in vivo* need to be done. In addition to phenolics content, HPm512 has a high protein content. Although not all quality characteristics are improved under similar conditions, it is worth pointing out improving phenolics accumulation may be beneficial to the accumulation of other nutrition components in wheat grains, such as carotenoids; significant positive correlation was observed between flavonoid and carotenoid content (15). Also, a higher number of DEMs were observed in mature HPm512 and ZM22 grains than immature grains. This may be due to the different synthesis rates of phenolic components in the late filling stage. Agronomic treatments at the later grain filling stage may be more effective for increasing phenolic levels than at the early stage (13, 30). Orientin and isoorientin were significantly and highly overexpressed in immature grains (30 DAA), indicating that HPm512 can be used for the production of food rich in bioactive substances even in immature grains. It is widely known that planting environment has a significant effect on grain phenolics content (10). We have to mention that although total phenolics content of HPm512 varied in different planting conditions, its relative abundance was significantly higher than that of control ZM22, which confirmed that HPm512 has potential health benefits.

In the phenylpropanoid biosynthetic pathway, conversion of phenylalanine to cinnamic acid by phenylalanine ammonia-lyase is the first step in phenolic biosynthesis. Generally, phenolic biosynthesis is correlated to structural genes, such as chalcone synthase, chalcone isomerase, flavanone3-hydroxylase, and regulatory genes, including some transcription factors (10). In this study, a higher number of genes involved in the accumulation of phenolics were down-regulated during the grain filling stage, which was consistent with the trend of phenolic expression levels during grain filling. Multiple copies of each gene are present in the hexaploid wheat genome (44). For example, 16 flavonol synthase genes were down-regulated, whereas 8 flavonol synthase genes were up-regulated during the grain filling stage. The differential expression of homologous genes may be correlated with the functional role of these genes in phenolic biosynthesis. Still, the finding suggests that flavonol synthase (TraesCS1A02G221200 and TraesCS1A02G320000) could play an important role in phenolics biosynthesis, and improving the expression of these genes may contribute to the accumulation of flavonol components. Its function needs further confirmation. Phenolic components commonly exist in glycosidic forms, and glycosyltransferase catalyzes glucosylation of various flavonoids. Chen et al. identified two candidate genes that could catalyze glucosylation and subsequent malonylation of various flavonoids (45). Wang et al. reported that altered expression levels of several glycosyltransferase genes were consistent with the accumulation of flavonoids in grains (19). In this study, the correlation analysis revealed that the expression levels of certain DEGs encoding glycosyltransferase were associated with the abundance of metabolites. Sequencing alignment of TraesCS7D02G198300 and TraesCS1D02G301700 indicated that these genes encode UDP-glucose:2-hydroxyflavanone C-glucosyltransferase, which catalyze the transfer of glucose from UDP-Glu to 2-hydroxynaringenin, forming the 2-hydroxy-6 (or 8)-C glucosylflavanone (46). Previous studies have shown that 2-hydroxyflavanones are intermediates in flavone biosynthesis, and isovitexin and vitexin are produced by elimination of H_2_O from 6 (or 8)-C-glucosyl-2-hydroxynaringenin (46, 47). Expression levels of these two genes were positively correlated with several phenolic components, including vitexin and isovitexin. The up-regulated expression of these glycosyltransferase genes in HPm512 may be related to the high abundance of vitexin and isovitexin.

Transcription factors are important regulators, which control plant's secondary metabolism by regulating the expression of genes involved in phenolic biosynthesis (48). Qu et al. reported that the transcription factor bZIP25, which is expressed in seed, could control flavonoid biosynthesis (49). In the current study, several bZIP transcription factors were found to be differentially expressed in HPm512 compared to ZM22, and their expression levels were positively correlated with the DEM accumulation. In addition, out of the differentially expressed transcription factors, the most common was AP2-ethylene-responsive transcription factor (ERF). Apart from their widely known roles in mediating plant responses to abiotic and biotic stresses, AP2/ERF members also play an important role in the regulation of plant growth and development. A previous study on medicinal plants revealed that ERFs are involved in the regulation of key steps in biosynthesis of pharmaceutically bioactive secondary metabolites (50, 51). In this study, the differential expression of many ERFs between the two wheat cultivars also suggests that they may be involved in regulating the accumulation of secondary metabolites. Exploring key regulatory genes and verifying their function can promote their successful applications in crop improvement programs. Furthermore, the function of other transcription factors and their interaction with ERFs cannot be ignored since multiple functions of AP2/ERF members are dependent on the interaction with other signaling pathways and regulatory networks (50).

## Conclusion

Phenolic content in wheat grains decreased during grain filling and showed a lower relative abundance in mature grains compared to immature grains. A total of 188 phenolic components, including 82 phenolic acids and 81 flavonoids, were significantly and differentially expressed during grain development. Apigenin glycosides were found to be the main flavonoid components in wheat grains. A higher number of up-regulated abundance flavonoid component in HPm512 mature grains may be the reason for its higher antioxidant activity. Transcription profiling revealed that the expression pattern of genes involved in phenolic accumulation was consistent with the trend of phenolic abundance during grain development. Integrated transcriptomics and metabolomics analysis revealed crucial information on the accumulation of phenolics in wheat grains. Different expression patterns of homologous genes also showed that there was multi-gene coordination in phenolic synthesis. Some identified differentially expressed genes, such as flavonol synthase (TraesCS1A02G221200 and TraesCS1A02G320000), and glycosyltransferase genes (TraesCS7D02G198300 and TraesCS1D02G301700) may play an important role in phenolics biosynthesis. We should like to point out that we have not verified the function of the identified key genes. The function and regulation mechanism of these genes needs to be gained in further studies.

## Data Availability Statement

The original contributions presented in the study are publicly available. This data can be found here: https://www.ncbi.nlm.nih.gov/geo/, GSE192732.

## Author Contributions

DM: methodology, formal analysis, data curation, and writing. BX: formal analysis and data curation. JF: investigation and data curation. HH: data curation and statistical analysis. JT and GY: supervision and resources. CW: supervision and editing. YX: conceptualization and supervision. All authors contributed to the article and approved the submitted version.

## Funding

This work was funded by the National Key Research and Development Program of China with grant 2016YFD0300404 and Scientific and Technological Project in Henan Province (222102110399).

## Conflict of Interest

The authors declare that the research was conducted in the absence of any commercial or financial relationships that could be construed as a potential conflict of interest.

## Publisher's Note

All claims expressed in this article are solely those of the authors and do not necessarily represent those of their affiliated organizations, or those of the publisher, the editors and the reviewers. Any product that may be evaluated in this article, or claim that may be made by its manufacturer, is not guaranteed or endorsed by the publisher.
