# Supplementary figures and images for: Dynamic Metabolomics and Transcriptomics Analyses for Characterization of Phenolic Compounds and Their Biosynthetic Characteristics in Wheat Grain
Source: Front Nutr. 2022 Feb 16;9:844337. doi: 10.3389/fnut.2022.844337 (PMC8888538; doi:10.3389/fnut.2022.844337)

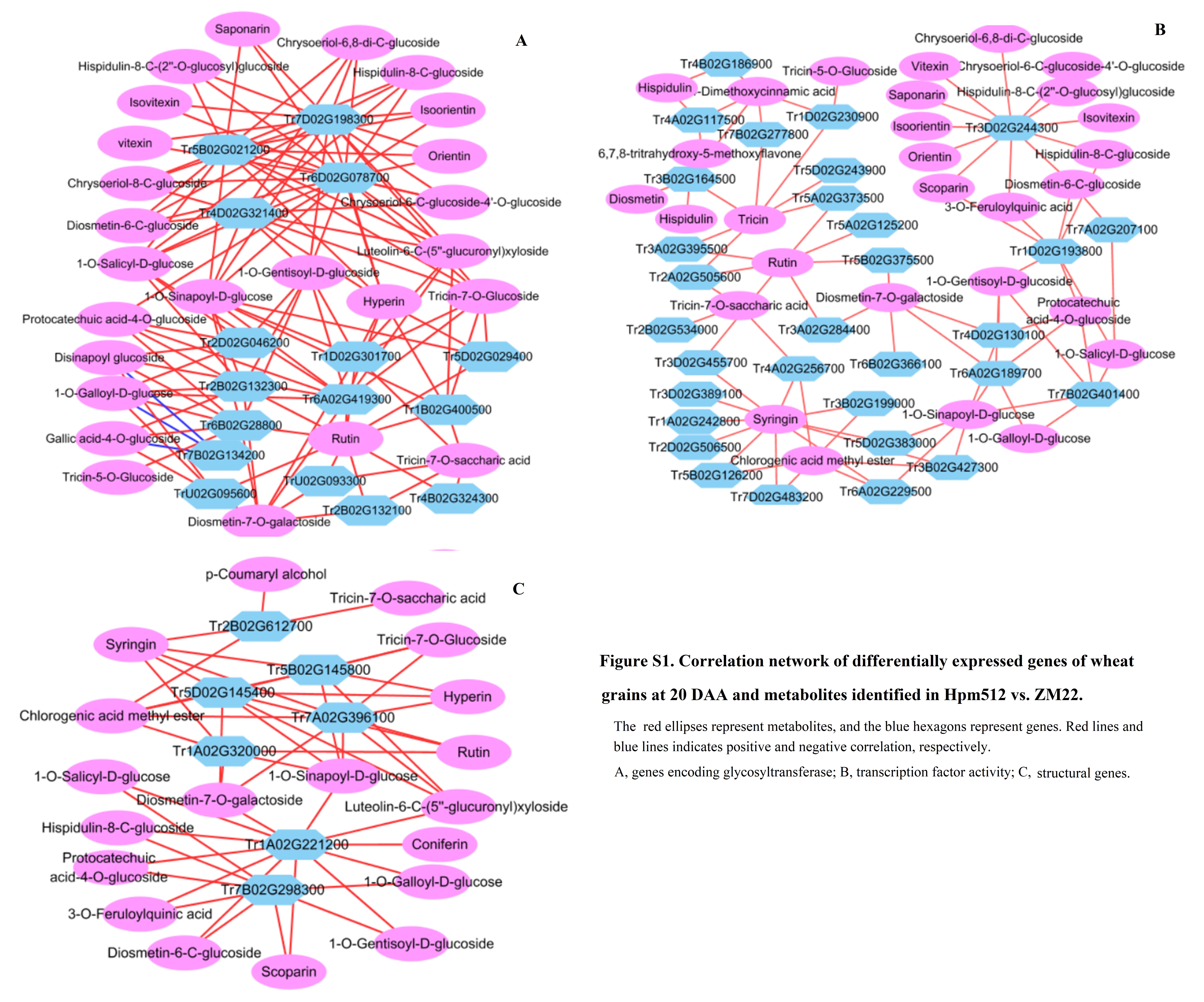

Supplement: Supplementary file 10 [file Image_1.JPEG]

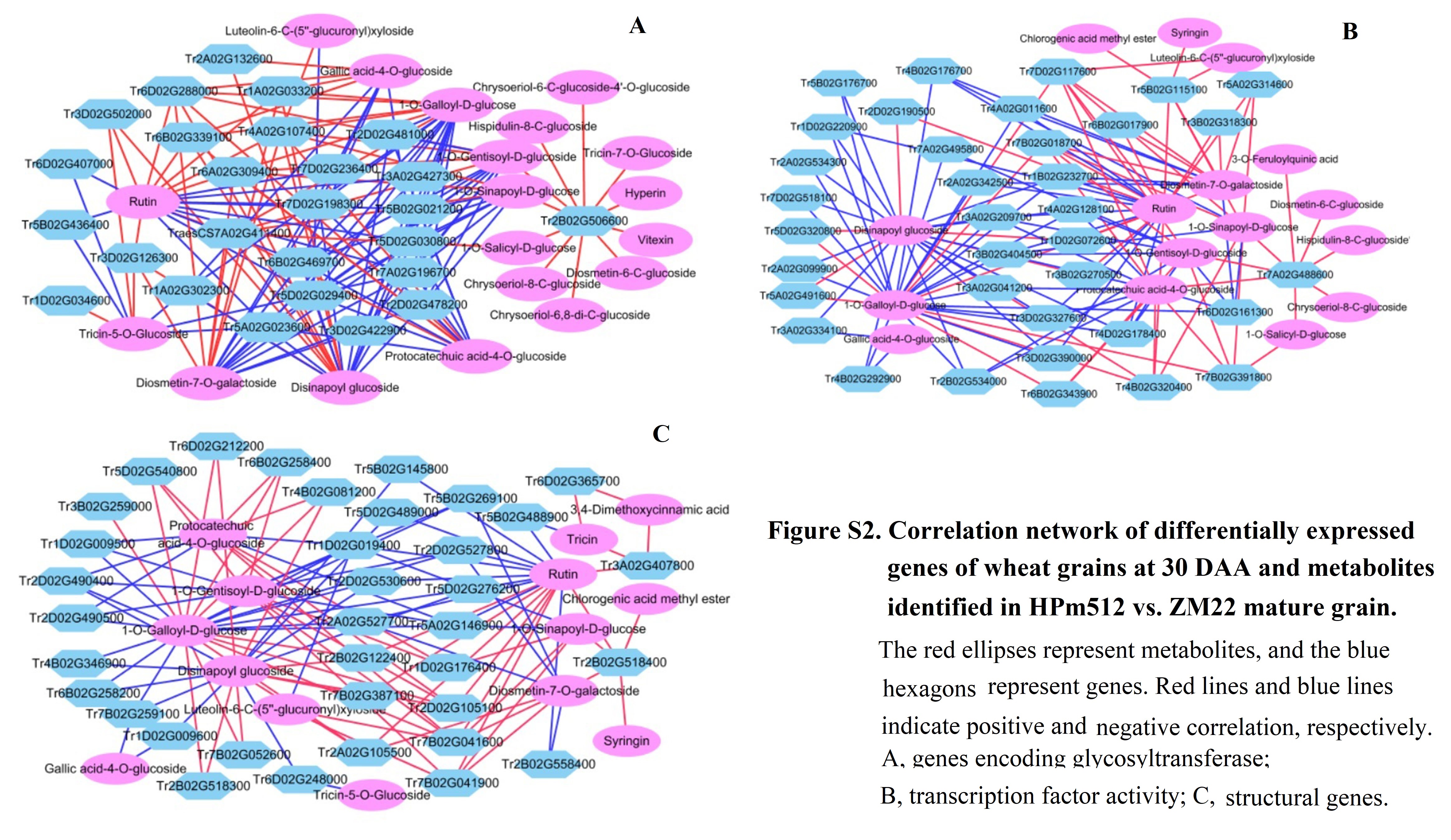

Supplement: Supplementary file 11 [file Image_2.JPEG]
